# The Institutional Library

**Published:** 1921-05-28

**Authors:** 


### THE INSTITUTIONAL LIBRARY.

**Standard Nomenclature of Diseases and Pathological Conditions, Injuries, and Poisonings, for the United States.** Department of Commerce Bureau of the Census. By SAM. L. ROGERS, Director. First edition. (Washington Government Printing Office. 1920.)

MEDICINE is not an exact science, and this fact is early grasped by the lay student of medical subjects as he finds that there is no uniformity of expression in medical terms. Authors of many of the systems of medicine have used their own judgment in the selection of names for diseases, so that each has in a sense a nomenclature of his own.

This American volume is an attempt to consolidate with our British nomenclature of diseases no less than seven others—namely, those of the American Army, Navy, Public Health Service, Expeditionary Forces, and three great American general hospitals.

To the names taken from these nomenclatures have been added terms from standard works on medicine and surgery, the Johns Hopkins Hospital Reports, and such medical dictionaries as Gould, Stedman, and Dorland. The book is divided into three sections, viz. (*a*) list of diseases and pathological conditions; (*b*) list of injuries, wounds, etc., by external causes; and (*c*) list of poisonings and intoxications. "Preferred terms," *i.e.* names which are to be used, are printed in bold-faced type and preceded by numbers. The first "preferred term" in the list of diseases beginning with the letter A has the number 1,001, the first preferred term under the second letter of the alphabet 2,001, and so on. Terms in the list of injuries and list of poisonings are similarly numbered, the letter A preceding the number in the case of injuries and the letter B in the case of poisonings.

On looking through the list of diseases one at once turns to rheumatism, a condition which caused us a good deal of anxiety in the matter of nomenclature during the war. According to the Royal College of Physicians there is no such thing—a man must have either rheumatic fever or myalgia. In this nomenclature rheumatism is "Arthritis, infectious, chronic," a doubtful improvement. Under poliomyelitis the anterior acute variety is alone recognised, and the posterior affection described in Osler and other text-books is only shown under the heading "Herpes Zoster." Normal parturition is recognised as a "disease," which, if inaccurate, will gladden the heart of those responsible for the statistics of maternity hospitals. Pappataci fever is shown, but the term Sandfly fever does not appear. Malarial cachexia, a useful term, long discarded in this country, finds a place in this volume, which reflects credit on Dr. William H. Davis, chief statistician for vital statistics of the Bureau of Census, under whose supervision it has been produced.

The Bureau emphasise the fact that it is a first edition intended to draw from physicians and all others interested free criticisms, which will help in making the next edition of greater value.

**Essentials of Medicine: a Text-book of Medicine.** By Charles Phillips Emerson, M.D. Illustrated by the author. Lippincott's Nursing Manual. (Philadelphia and London: J. B. Lippincott Co. 12s. 6d. net. Fourth edition, revised.)

THIS handy volume, very clearly written and well arranged, is intended (*a*) for students beginning a medical course, (*b*) for nurses, (*c*) for all others interested in the care of the sick. Opinions differ strongly as to the advisability of setting nurses to study medical works. In our judgment much harm may be done by a cursory examination into diseased conditions by those who undertake the care of the sick under medical practitioners; and we should be sorry indeed to see persons who were neither students nor nurses absorbed in such studies. But there are occasions when resort to a manual of this description is called for, and its presence in the reference library would be useful. Dr. Emerson has avoided the blunder of giving directions which might lead to self-medication or prescription by nurses, and much of what he says on the reasons for the use of certain remedies would prove a groundwork for lectures to probationers.

**The Medical Register, 1921. The Dentists' Register, 1921.** (Published for the General Medical Council by Constable & Co., Ltd. 21s., 3s. 4d.)

THE table headed as showing the number of persons whose names were entered in, added to, or removed from the Medical Registers for the several years from 1893 to 1919 inclusive is better than it describes itself to be, for it actually extends to 1920. In that year a total of 1,457 names were added to the Register, of which 457 are in England, 393 in Scotland, 264 in Ireland, 333 Colonial, and 10 foreign. Three names were restored and 1,241 names removed, of which 751 are due to death. The total number on the Register at December 31, 1920, was 44,761, which shows an increase of nearly 700 on the average for the last five years, viz. 44,099. The average for the last twenty-four years was 40,074. The number of names added to the Dentists' Register for 1921 is 194 British, whilst 71 were removed, 66 being by death. The present total number on the Dentists' Register is 5,610, the average for the previous five years being 5,502.

**Clinical Disorders of the Heart Beat.** By THOMAS LEWIS, M.D., F.R.S., D.Sc., F.R.C.P., C.B.E. (Fifth edition. Pp. 128. 8s. 6d. net. Messrs. Shaw & Sons.)

DR. THOMAS LEWIS is such a well-known leader in cardiological research that his books need no special introduction from the reviewer. This little book—in its fifth edition in eight years—is a companion volume to two other useful books—"Clinical Electrocardiography" and "The Soldier's Heart and the Effort Syndrome." In this work the different types of cardiac irregularity are carefully explained, and numerous polygraphic tracings are fully analysed to bear out and illustrate the nature of the lesions discussed.

The tracings shown are full of interest, and demonstrate still further—if need there be—the great uses of the polygraph. This is an instrument simple to understand and easy to work, and it is hoped that in time it will be used in the investigation of a cardiac case as frequently as the stethoscope is now used. This book is valuable in the rush of our present-day work because of its size, the wealth of detailed information in a small space, and its essentially practical nature.

The electrocardiagraph is not mentioned, since Dr. Lewis has described this instrument fully in his other works.

